# Demographic and Clinical Features of Dengue Fever in Pakistan from 2003–2007: A Retrospective Cross-Sectional Study

**DOI:** 10.1371/journal.pone.0012505

**Published:** 2010-09-13

**Authors:** Erum Khan, Mehreen Kisat, Nabil Khan, Amna Nasir, Salma Ayub, Rumina Hasan

**Affiliations:** 1 Department of Pathology and Microbiology, Aga Khan University Hospital, Karachi, Pakistan; 2 Medical College, Aga Khan University Hospital, Karachi, Pakistan; University of California Los Angeles, United States of America

## Abstract

**Background:**

Demographic features of dengue fever have changed tremendously in Pakistan over the past two decades. Small scale studies from all over the country have reported different aspects of individual outbreaks during this time. However, there is scarcity of data looking at the overall trend of dengue virus infection in the country. In this study, we examined annual trends, seasonality, and clinical features of dengue fever in the Pakistani population.

**Methods:**

Demographic information and dengue IgM status of all patients tested for dengue IgM antibody at Aga Khan University Hospital from January 2003 to December 2007 were analyzed to look for trends of IgM-positive cases in Pakistan. In addition, clinical and biochemical parameters were abstracted retrospectively from medical records of all patients hospitalized with IgM-proven dengue fever between January 2006 and December 2007. These patients were categorized into dengue fever and dengue hemorrhagic fever according to the WHO severity grading scale.

**Results:**

Out of a total of 15040 patients (63.2% male and 36.8% female), 3952 (26.3%) tested positive for dengue IgM antibody. 209 IgM proven dengue patients were hospitalized during the study period. During 2003, IgM positive cases were seen only during the months of July-December. In contrast, such cases were detected throughout the year from the 2004–2007. The median age of IgM positive patients decreased every year from 32.0 years in 2003 to 24.0 years in 2007 (p<0.001). Among hospitalized patients, nausea was the most common presenting feature found in 124/209 (59.3%) patients. Children presented with a higher median body temperature than adults (p = 0.010). In addition, neutropenia was seen more commonly in children while raised serum ALT levels were seen more commonly in adults (both p = 0.006). While a low total white cell count was more common in patients with dengue fever as compared to Dengue Hemorrhagic Fever (p = 0.020), neutropenia (p = 0.019), monocytosis (p = 0.001) and raised serum ALT level (p = 0.005) were observed more commonly in the latter group.

**Conclusions:**

Dengue virus is now endemic in Pakistan, circulating throughout the year with a peak incidence in the post monsoon period. Median age of dengue patients has decreased and younger patients may be more susceptible. Total and differential leukocyte counts may help identify patients at risk of hemorrhage.

## Introduction

Dengue Virus (DV) is an enveloped, single-stranded, positive RNA virus and a member of the family *Flaviviridae*, genus *flavivirus*. There are four antigenically related but distinct serologic subtypes; DV-1, DV-2, DV-3 and DV-4. An estimated 50-100 million cases of Dengue Fever (DF) and about 250,000–500,000 cases of Dengue Hemorrhagic Fever (DHF) occur every year [1]. In South East Asia, the average number of cases of DHF per year has increased from 10,000 in the 1950s to over 200,000 in the 1990s. Thus, dengue virus remains a major cause of morbidity and mortality in tropical areas [2].

Epidemic dengue fever was common in Asia and Pacific throughout the twentieth century [3]. Dengue virus made its route geographically into Asia through South Asian countries [4]. In Asia, the first outbreak of DHF began in the 1950s in the Philippines and Thailand. However, in the next 20 years, the disease spread throughout South East Asia and by the mid 1970s, DHF was the leading cause of hospitalization and death among children in this region. Thereafter in the 1980s and 1990s, dengue transmission intensified with regular epidemics every 3–5 years in hyperendemic areas [5].

In Pakistan, the first confirmed outbreak was due to serotype DV-2 reported in 1994 by Aga Khan University Hospital (AKUH) [6]. Thereafter, sporadic cases of DHF continued to be reported from different parts of the country. Akram et al reported the presence of DV-1 and DV-2 in the sera of children with undifferentiated fever [7]. In addition, Paul et al reported in 1998 that an outbreak of dengue fever in the Balochistan province was due to co-circulation of DV-1 and DV-2 [8].

During September to December 2005, three major hospitals in Karachi had a sudden increase in the number of patients with DHF [9]. Genotyping of selected samples from the early part of the outbreak revealed the presence of DV-3. This epidemic was probably a consequence of the introduction of DV-3 in a population with prior sensitization to DV-1 and DV-2 resulting in severe disease. Thereafter, Pakistan experienced its largest and most severe outbreak of DHF in 2006 and DV-2 and DV-3 were identified as the predominant serotypes [10].

The demographic features of DF and DHF have changed tremendously in South East Asia and Pakistan over the past two decades. Small scale studies from all over the country have reported different aspects of major individual outbreaks during this time. However, there is scarcity of data looking at the overall trend of DV infection in the country. We maintained a data and serum bank of all the patients tested for the presence of dengue IgM antibodies from 2003–2007. In the current study, we have looked at annual trends and demographics of dengue infections in the Pakistani population over five years. In addition, we have also evaluated clinical and laboratory features of patients hospitalized with dengue fever during 2006–2007.

## Methods

### Ethics Statement

The study protocol was approved by Ethical Review Committee at Aga Khan University Hospital. No interventions or any sort of study-related patient interaction was involved. In addition, all data was analyzed anonymously and hence, informed consent was not required.

### Location and sampling

**Figure 1:**
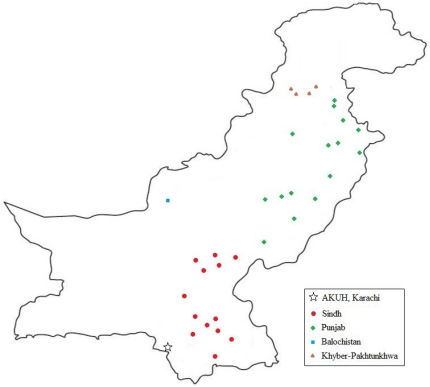
Geographical distribution of cities with laboratory collection points in Pakistan. This map presents the geographical distribution of cities with laboratory collection points from where samples used in this study were received. Colours denote the four provinces of Pakistan as described in the legend. Aga Khan University Hospital (AKU, Karachi) was the study center.

AKUH is a 550-bed tertiary care hospital located in Karachi, Pakistan. The clinical laboratory has a unique network of clinical sample collection units spread through out the country. Samples were received from 125 collection sites including the main hospital site, 71 sites within Karachi and 44 sites in the rest of Pakistan. Distribution of samples received and geographical location of sites has been described in Figure 1. Demographic data from all patients tested for dengue IgM antibody between January 2003 and December 2007 at AKUH was included in this study. In addition, medical records of all patients with IgM proven dengue who were hospitalized for treatment at AKUH from 2006–2007 was reviewed retrospectively for clinical and biochemical data. Hospitalized patients were categorized into Dengue Fever, Dengue Hemorrhagic Fever and Dengue Shock Syndrome according to the World Health Organization (WHO) severity grading scale [11].

Dengue IgM antibodies were detected using the Calbiotech Inc. ELISA test system (Catalog No. DE051M). This is a commercial Enzyme-Linked ImmunoSorbent Assay for detecting IgM antibodies against dengue virus in human serum or plasma. Optical density (OD) was read at dual wavelength with reference filter of 600–650nm. Antibody Index was calculated using the OD value and the cut-off value (Calibrator OD × Calibrator Factor). Antibody index of >1.1 was considered positive for acute dengue infection and those between 0.9 and 1.1 were included in borderline positive category. Hence, samples with indexes below 0.9 were concluded as negative for dengue infection.

### Data management and statistical analysis

The blood indices were initially measured on a continuous scale and then categorized on the basis of clinically meaningful cutoffs. Thrombocytopenia was defined as a platelet count <150,000 cells/mm^3^ blood. A hematocrit value >48% was considered raised. Similarly, leucopenia was defined as a white cell count <4000 cells/mm^3^, neutropenia as neutrophils <40%, lymphocytosis as lymphocytes >45% and monocytosis as monocytes >10%. Alanine aminotransferase (ALT) was considered raised if >55 and >33 IU/L for males and females, respectively. Aspartate aminotransferase (AST) was defined as raised if >46 and >32 IU/L for males and females, respectively.

Statistical Package for Social Sciences (SPSS) version 15.0 (Chicago, IL) was used for data entry, processing and statistical analysis at the end of the study period. Descriptive statistics were calculated for all relevant variables. Since age and laboratory measurements were not normally distributed (Shapiro-Wilk p<0.05), median ± interquartile range has been reported. Differences between several groups of patients were evaluated using Chi-square test or Fisher's Exact test for categorical variables and Mann-Whitney U test for continuous variables. Odds Ratios (OR) were calculated to evaluate the relationship between two categorical variables. P-values less than 0.05 were considered significant.

## Results

### Demographic Findings

A total of 15040 serum samples were tested for dengue IgM antibodies during the study period (2003–2007). This included 9500 males (63.2%) and 5540 females (36.8%). 11917 patients (90.9%) were from Karachi while 1199 patients (9.1%) were from other cities of Pakistan (**Figure 1**) The median age of the patients was 24.0±20.0 years. There were 11041 (73.6%) adults and 3955 (26.4%) children (aged less than equal to 15 years). The total number of people tested for Dengue IgM increased progressively until 2006 and then declined in 2007.

**Figure 2:**
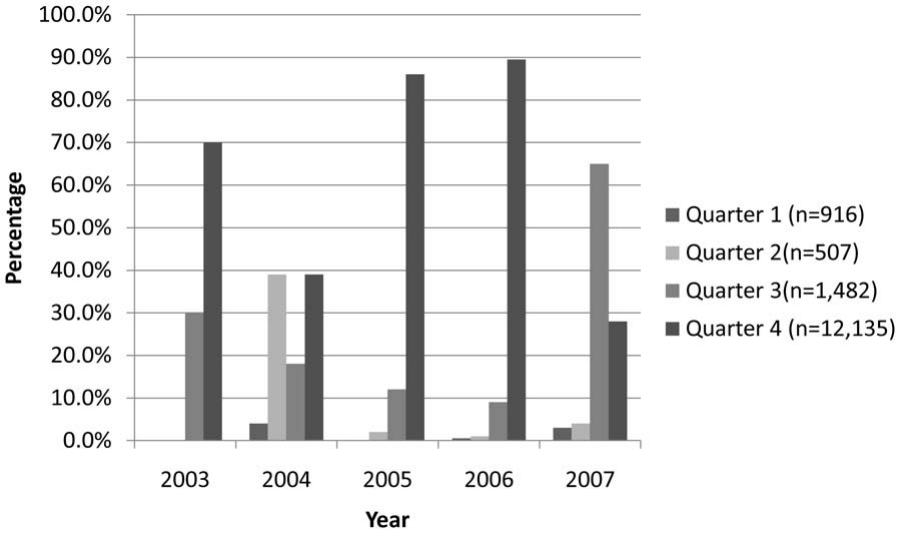
Quarterly distribution of the percentage of Dengue IgM positive cases by year. In 2003, Dengue IgM positive cases were only observed to appear in the months of July- December. This trend changed from 2004–2007, when IgM positive cases were detected throughout the year. Dengue IgM positive cases were most prevalent in the fourth quarter (October–December) from the years 2003 to 2006.

Of the 15040 patients tested, 3952 samples (26.3%) were positive for Dengue IgM antibody. There was no significant difference in seropositivity between samples collected within or outside Karachi (p = 0.221). The percentage of patients demonstrating IgM seropositivity increased from 28.3% in 2003 to 35.9% in 2004 and then decreased progressively from 2005 to 2007. The trends observed in percentage of patients with dengue IgM seropositivity have been illustrated quarter-wise in **Figure 2**. During 2003, IgM positive cases were only observed in the months of July - December. However, such cases were detected throughout the year from 2004–2007. In addition, the highest proportion of seropositivity was seen in the last three months of the year from 2003–2006. However, in 2007, the highest proportion of such cases was observed in the third quarter of the year.

61% (n = 2403) of total patients who tested positive for Dengue IgM during all five years were male. Although not statistically significant, this gender distribution evolved from 2003 to 2007 such that the proportion of females amongst IgM positive cases increased (p = 0.253). On the whole, the odds of seropositivity over all 5 years were higher in women than in men (95% CI OR: 1.07, 1.25; p<0.001).

**Figure 3:**
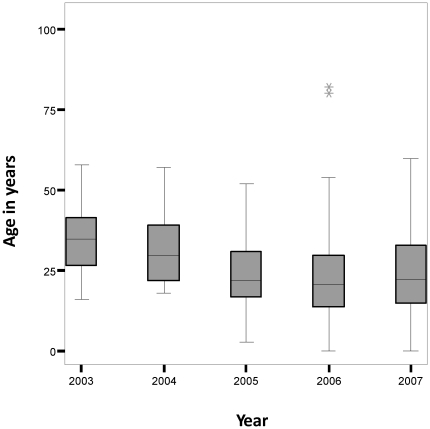
Median age of patients from 2003–2007. The median age of patients has decreased between the years 2003 and 2007.

The median age of the dengue patients has decreased over the study period from 32.0 years in 2003 to 24.0 years in 2007 (p<0.001, d.f. = 4, see **Figure 3**). While the highest proportion of seropositivity was observed in patients aged 26–40 years in 2003 and 2004, this trend shifted to patients aged 11–25 years in 2005–2007 (see **Table 1**). In addition, the median age of female dengue patients was significantly lower than males (24.2 years compared to 25.9 years respectively: p<0.001).

**Table 1:** Distribution of dengue IgM positive cases according to age groups from 2003–2007.

	Number of patients in each age group(age in years) – n(%)^1^							
Year	< = 2	3–10	11–25	26–40	41–55	56–70	> = 71	Total
**2003**	0 (0.0)	0 (0.0)	7 (19.4)	21 (58.3)	5 (13.9)	2 (5.6)	1 (2.8)	36
**2004**	0 (0.0)	0 (0.0)	8 (34.8)	9 (39.1)	3 (13.0)	2 (8.7)	1 (4.3)	23
**2005**	0 (0.0)	4 (2.6)	83 (53.5)	43 (27.7)	18 (11.6)	7 (4.5)	0 (0.0)	155
**2006**	77 (2.4)	357 (10.9)	1475 (45.2)	944 (28.9)	306 (9.4)	98 (3.0)	9 (3.0)	3266
**2007**	9 (2.0)	63 (13.9)	202 (44.7)	116 (25.7)	47 (10.4)	13 (2.9)	2 (4.0)	452
**Total**	86 (2.2)	424 (10.8)	1775 (45.1)	1133 (28.8)	379 (9.6)	122 (3.1)	13 (3.0)	3932

1 Percentage was expressed using total number of dengue IgM positive patients for each year as denominator.

### Clinical and Laboratory Findings

A total of 209 patients including 132 males (63.2%) and 77 females (36.8%) with a median age of 25±17 years were hospitalized at AKUH during the defined study period.

Within this cohort, the three most common presenting features were nausea in 124 patients (59.3%), rash in 76 patients (36.4%) and myalgia in 54 patients (25.8%) followed by hemorrhage in 38 patients (18.2%), diarrhea in 34 patients (16.3%), cough in 23 patients (11.0%) and headache in 23 patients (11.0%). Hemoglobin levels less than 10 mg/dL were observed in 27/182 patients (14.8%). Total leukocyte count was decreased in 75/186 patients (40.3%). In the differential leukocyte count, 52/160 patients (32.5%) had monocytosis, 55/173 patients (31.8%) had neutropenia and 41/171 patients (24.0%) had lymphocytosis. 158/199 patients (79.4%) had thrombocytopenia at presentation. AST was raised in 93/106 patients (87.7%) and ALT was raised in 91/145 patients (62.8%). Males had a higher hematocrit (41.5±6.8%) as compared to females (36.8±6.2%) at the time of admission (p<0.001).

There were 81% (169/209) adults (31.9±12.9 years) and 19% (40/209) children (9.3±4.5 years) in this sample. Body temperature was significantly higher in children (38.0±2.0°C) as compared to adults (37.6±1.0°C; p = 0.010). Neutropenia was also seen more commonly in children (p = 0.006). ALT levels were raised in 83 out of 123 (64.5%) adults as compared to 8 out of 22 (36.4%) children (p = 0.006). However, no significant difference in AST levels at presentation was seen.

Dengue fever was diagnosed in 161/206 patients (99 male, 62 female) and dengue hemorrhagic fever was diagnosed in 40/206 patients (30 male, 10 female, p = 0.077). 5/206 patients were classified as Dengue Shock Syndrome and were excluded from comparative analysis due to their statistically inadequate number. Sociodemographic, clinical and laboratory features of these patients with DF and DHF have been described in **Table 2**. 18.6% patients with dengue fever and 17.5% patients with dengue hemorrhagic fever were below the age of 15 years.

**Table 2:** Characteristics of patients with dengue fever (DF) and dengue hemorrhagic fever (DHF)- n(%).

Characteristic	DF^1^(%)	DHF^1^(%)	N	p-value^2^
**Demographics**				
Age (median ± interquartile range)	26.0±19.0	24.5±18.0	201	0.905
Gender				
Male	99(61.5)	30(75.0)	201	0.077
Female	62(38.5)	10(25.0)		
**Clinical Presentation**				
Fever	159/161(98.8)	39/39(100.0)	200	0.647
Nausea and/or vomiting	93/161(57.8)	27/40(67.5)	201	0.173
Rash	44/161(27.3)	31/40(77.5)	201	<0.001
Abdominal Pain	6/86(7.0)	1/11(9.1)	97	0.582
Diarrhea	26/161(16.2)	5/40(12.5)	201	0.384
Myalgia	46/161(28.6)	6/40(15.0)	201	0.056
Headache	21/161(13.0)	2/40(5.0)	201	0.120
Cough	22/161(13.7)	1/40(2.5)	201	0.033
Hemorrhage	6/161(3.7)	29/40(72.5)	201	<0.001
Temperature (median)	38.00±2	37.00±1	177	0.014
**Laboratory Findings**				
Thrombocytopenia at presentation	116/151(76.8)	36/40(90.0)	191	0.047
Received platelet transfusion	25/90(27.8)	13/32(40.6)	122	0.131
Low Hemoglobin	16/137(11.7)	8/37(21.6)	174	0.102
Hematocrit level on admission (median)	39.50±6.75	40.40±8.80	167	0.607
Leukopenia	64/141(45.4)	9/37(24.3)	178	0.015
Neutropenia	35/133(26.3)	16/34(47.1)	167	0.018
Lymphocytosis	30/132(22.7)	7/33(21.2)	165	0.529
Monocytosis	32/122(26.2)	19/33(57.6)	155	0.001
Raised ALT	63/110(57.3)	27/32(84.4)	142	0.004
Raised AST	68/79(86.1)	25/26(96.2)	105	0.147

1 Reported as number of individuals with finding/number of individuals in the category. For example, diarrhea is reported as 26/161 under Dengue Fever implying that 26 patients had diarrhea out of 161 total Dengue Fever patients. DF – Dengue Fever, DHF – Dengue Hemorrhagic Fever.

2 p-value was calculated using Mann-Whitney U test for age, temperature and hematocrit. Chi-square/Fisher's exact test were used for all other categorical variables.

**Figure 4:**
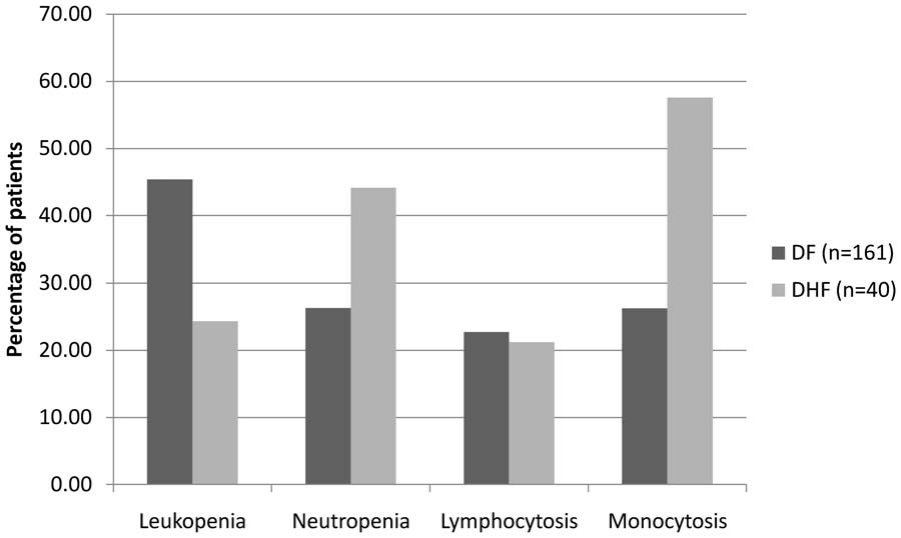
Comparison of total and differential leukocyte counts between dengue fever(DF) and dengue hemorrhagic fever(DHF). Patients with DF were more likely to have leukopenia (p = 0.015) with a normal differential count while patients with DHF were more likely have a normal TLC count, with neutropenia (p = 0.018) and monocytosis (p = 0.001).

31 out of 40 patients with DHF developed a rash as compared to 44 out of 161 patients with DF (p<0.001). 36 out of 40 patients with DHF had thrombocytopenia on presentation compared to 116 out of 151 patients with DF (p = 0.047). Leukopenia was seen more commonly in patients with DF as compared to DHF (p = 0.015). On the other hand, neutropenia and monocytosis were seen more commonly in patients with DHF (p = 0.019 and p = 0.001 respectively; see **Figure 4**). Median ALT levels were significantly higher in patients with DHF as compared to the patients with DF (p = 0.011).

## Discussion

Dengue virus presently threatens half of the world's population and is an important public health problem in many tropical regions of the world [12]. In the last three decades, the demographic and clinical features of dengue infections have changed rapidly. Pakistan experienced the first major outbreak of Dengue in 2004 and the serotype identified was DV-2 [6]. Thereafter, several studies from Pakistan and other endemic areas reported DV-1 and DV-2 to be the predominant serotypes in circulation [7], [8]. This was followed by the introduction of DV-3 in our population in 2005 resulting in another epidemic [9]. During the most recent epidemic in 2006, the genotypes DV-2 and DV-3 were found to be prevalent in Pakistan [10].

The epidemics of dengue have been commonly associated with the rainy season [13]. The peak incidence of dengue fever was reported to occur from August to October in Pakistan [14]. Similarly, this study found the highest proportion of IgM positive dengue patients in the last two quarters of 2006 and 2007, an observation also shared by another South Asian study [15]. While the first IgM positive cases were detected in the month of July in 2003 in our study, such cases were prevalent throughout the year from 2004–2007. This is consistent with another recent study from Hong Kong where dengue cases were reported all year round [16]. Similarly, Tripathi et al reported that dengue transmission occurred round the year in Lucknow region in India with peak incidence in the post-monsoon season [17]. This trend may be explained by the hyper-endemicity of the virus and the co-circulation of all four serotypes. Factors responsible for maintenance of dengue fever throughout the year need to be identified and addressed in future studies.

There is conflicting data on the shift in median age of dengue virus. Several studies from Asia using surveillance data report increasing age of effected patients. In Indonesia, data from 1975 to 1984 showed an increase in incidence rates among young adults in Jakarta as well as in the provincial areas [18]. In contrast, a decrease in median age of patients affected with dengue from 2003–2007 was seen in this study. The most common age group affected in the recent years was 11–25 years. A progressive increase in the proportion of children affected with dengue was also observed. Not a single child was affected in 2003 and 2004 but number of children testing positive for dengue IgM increased dramatically between 2005 and 2007. Since the DV-2 strain has been circulating in the Pakistani population since 2004, the observed shift in median age may be explained by adults acquiring immunity due to earlier infections [6]. This observation also suggests that dengue fever should be considered as a differential in children presenting with febrile illnesses in this region. In addition, public health measures aimed at the control of dengue also need to consider the increasing number of affected children.

Most developing countries have epidemics of febrile illnesses which can be confused with dengue fever [19]. A recent review of 15 published studies was unable to make any conclusions on the signs and symptoms that can clinically distinguish dengue from other febrile illnesses [20]. However, Srikiatkhacorn et. Al reported a high specificity (99%) of the WHO criteria in a prospective sample of dengue cases in Thailand [21]. The majority of hospitalized patients in this study presented with nausea, rash and myalgia in decreasing order of frequency. The reported frequency of rash in dengue fever ranges from 50–66%[22]. Recently, Premaratna et al demonstrated a rash in 95% of the patients using a hand impression technique in a study conducted at a teaching hospital in Sri Lanka [23]. In view of this, it is suggested that evaluation of rash in patients presenting with short duration fever may help in the diagnosis of dengue fever.

Most patients in this study had thrombocytopenia and deranged liver enzymes at the time of admission. In addition, median ALT levels were significantly higher in patients with DHF as compared to those with DF and in adults as compared to children. Liver injury is a common finding in dengue infections and it is mediated by direct infection of hepatocytes and Kupffer cells [24], [25]. Recently, Luiz et al suggested the use of markers such as ALT and AST as parameters to evaluate severity in patients with dengue fever [24]. Since grossly elevated liver enzymes are known to be an early warning sign for severe disease and clinical bleeding, vigorous follow up in such patients is warranted [25].

Early identification of patients with dengue at risk of developing hemorrhage is an important clinical objective [20]. It was observed that leukopenia was significantly more common in patients with DF. On the other hand, raised neutrophil and monocyte counts were more common in patients with DHF. Thus, total and differential leukocyte count may be possible markers that distinguish DF and DHF. However, additional prospective studies are needed to establish an algorithm based on clinical and laboratory features that can be validated and generalized to identify individuals at risk of hemorrhage in the early stages of illness.

Limitations of this study include its reliance on a convenience sample from a laboratory serum bank for demographic data and therefore, the proportion of dengue IgM positive patients may be overestimated due to a selection bias. While the reported proportions should not be mistaken for community prevalence of dengue, this strategy remains appropriate for comparison of demographic trends over the study period because sampling conditions have not changed significantly over these years. In addition, laboratory samples were received through a network of 125 collection points spread throughout the country. It may be argued that awareness of the population regarding dengue increased over the study period. However, it implies that an increase in the total number of individuals tested each year would be observed. No such trends of a yearly increase in total number of individuals tested or proportion of dengue positive individuals was observed and hence, increase in public awareness about dengue does not seem to have influenced our results.

### Conclusion

The results of this study describe the demographic trends of dengue infections in Pakistan. Dengue virus is now endemic in the country, circulating throughout the year with a peak incidence in the post-monsoon period. Median age of dengue patients has decreased and younger patients may have become more susceptible. Total and differential leukocyte count may be useful for identification of patients at risk of hemorrhage and their utility needs to be studied further.
